# Paget Disease as Common Pitfall on PET with Different Radiopharmaceuticals in Oncology: Not All That Glitters Is Gold!

**DOI:** 10.3390/jcm11185372

**Published:** 2022-09-13

**Authors:** Francesco Dondi, Domenico Albano, Giorgio Treglia, Francesco Bertagna

**Affiliations:** 1 Division of Nuclear Medicine, ASST Spedali Civili di Brescia, 25123 Brescia, Italy; 2 Division of Nuclear Medicine, Università degli Studi di Brescia and ASST Spedali Civili di Brescia, 25123 Brescia, Italy; 3 Clinic of Nuclear Medicine, Imaging Institute of Southern Switzerland, Ente Ospedaliero Cantonale, 6500 Bellinzona, Switzerland; 4 Department of Nuclear Medicine and Molecular Imaging, Lausanne University Hospital and University of Lausanne, 1015 Lausanne, Switzerland; 5 Faculty of Biomedical Sciences, Università della Svizzera Italiana, 6900 Lugano, Switzerland

Paget’s disease (PD) is a benign but chronic condition characterized by the presence of abnormal homeostasis and remodeling of the bone, resulting in high osteoblastic and osteoclastic activity. Commonly, this disease affects 3–4% of the population over the age of 40 [1,2,3].

The exact etiology of PD is not clearly known; however, some contributory elements are thought to be familial predisposition and viral infections. Approximately 15–40% of people affected by PD have a positive familiar anamnesis, and an increased risk associated with human leukocyte antigen DR2 (HLA-DR2) serum marker has been proposed, suggesting the possible presence of a genetic susceptibility. The theory of viral origin of PD is supported by the presence of giant osteoclasts with intranuclear inclusion bodies in bone affected by the disease, which are findings also reported in viral infections. However, a large variety of other causes for the development of PD have also been proposed, including connective tissue disease, vascular disease, autoimmune disorder and metabolic disease related to increased parathyroid hormone levels [2].

PD is characterized by the presence of abnormal bone remodeling, and, classically, the presence of three distinctive phases has been described, although in the clinical reality they represent a single continuum: the lytic phase (with osteoclast predominance), the mixed phase (with osteoblasts that begin to predominate on osteoclastic activity) and the blastic phase (with a gradual decline in osteoblastic activity) [1,2,3]. These different phases can be present in the same patient, depending on both widespread osseous involvement and the variable progression rate of single sites. However, the final step is the presence of a thickened and disorganized trabecular bone structure [2].

The clinical presentation of PD can be very heterogeneous, with the absence of symptoms characterizing the initial stages in 20% of patients. In asymptomatic patients, the disease is usually incidentally detected, and, in such cases, it is important not to confuse its imaging appearance with those of other, similar diseases affecting the bones, including bone tumors. Complications of PD are related to the effects of osseous weakening, such as deformity and fractures, but can also include arthritis or neurological symptoms. Skeletal symptoms can include pain, tenderness, increased warmth, increased bone size, deformities and decreased range of motion. Neuro-muscular symptoms are consequences of bone enlargement near neural foramina or canals that can lead to the mechanical compression of nervous structures, resulting in deafness, visual abnormalities, weakness, paralysis and incontinence [2].

Regarding the localization of the disease, PD predominantly affects the axial skeleton, with the pelvis, the spine and the skull being the most commonly affected sites. Proximal segments of long bones are also frequently involved in the disease, in particular the femur in 25–35% of the cases. Furthermore, sites that are less commonly involved can include the shoulder girdle and forearm. The ribs, fibula, hands, feet, calcaneus, patella and tibial tubercle are sites that are rarely involved in the disease. Furthermore, the monostotic form of PD, meaning that only a single skeletal site is involved by the disease, is more often seen in the axial skeleton, although any site can be the sole region of involvement. When multiple sites are involved in the disease, polyostotic PD is present, and this particular form is more frequent than monostotic one, tends to have right-sided predominance and usually involves the lower extremities. Interestingly, pelvic involvement is often asymmetric, and appendicular involvement is frequently unilateral [2].

Regarding therapy, multiple agents have been proposed for the treatment of PD, including calcitonin, biphosphonates and mithramycin. The main goals of the therapy are the control, the reduction and the alleviation of pain, rather than the resolution of the disease and the return to a normal bone organization [2].

The diagnosis of PD is mainly based on the radiological evaluation of the skeleton, but blood tests and urine laboratory values can also help. The high amount of osteoclastic and osteoblastic changes in the bones can be underlined by the presence of elevated serum levels of alkaline phosphatase (ALP), in particular during the reparative phases of the disease. Increased serum and urine levels of hydroxyproline, related to an increased rate of bone resorption, can also be present in the lytic phase. Furthermore, serum levels of calcium and phosphate are usually normal in patients affected by PD, but 10% of them may develop secondary hyperparathyroidism and hypercalcemia related to the aggressive bone remodeling present in PD [2].

As mentioned previously, radiological evaluation of the bone is mandatory to reach a final diagnosis of PD, since plain radiograms (X-ray) and computed tomography (CT) are able to recognize the presence of both early lytic and late blastic phases [4]. X-ray is the mainstay for diagnosis of non-complicated forms of the disease. The early lytic phase is characterized by the presence of osteolysis in X-ray images; however, the majority of cases of PD are diagnosed in the mixed phase. In this phase, radiographs can underline the coarsening and thickening of the trabecular pattern. The mixed phase may progress to the blastic one, which is characterized by the presence of sclerosis with obliterating areas. Regarding CT, pagetic bone in the osteolytic phase may show osteolytic lesions, while in the intermediate and quiescent phases, it can show cortical and trabecular thickening. However, it is not uncommon to incidentally discover the presence of PD on CT images obtained for other reasons. Furthermore, areas of sclerosis may be seen in the blastic phase of the disease, and the marrow space in PD can often reveal fat attenuation. Non-complicated forms of disease show no evidence of cortical destruction or any soft-tissue mass [1,2,3].

Bone scintigraphy is also used for the assessment and the follow-up of PD [2,5,6,7,8,9,10,11,12,13,14,15], and, in particular, 3-phase bone scintigraphy can show blood-flow, blood-pool and metabolic bone imaging, highlighting increased radionuclide uptake in the region of abnormal bone in all three phases of the disease. However, such imaging modality is a sensitive but not specific examination. Areas of abnormal radionuclide uptake are usually elongated, reflecting the distribution of the disease rather than the characteristic circular abnormality more typical of metastatic disease or myeloma. Since scintigraphy is more sensitive to changes in vascularity, the hypervascular nature of PD is often demonstrated as increased radiopharmaceutical uptake, which may be detected even before the typical radiographic lucency. Sometimes, bone scintigraphy and X-ray imaging can demonstrate different findings, and it is important to recognize this spectrum of disparity. The various complications associated with PD are usually not differentiated with bone scintigraphy. In the past, bone scintigraphy was also used to assess the effects of therapy on PD, defined as the progressive reduction in the degree of radiopharmaceutical uptake during treatment. However, this imaging modality is no longer used for this purpose because of its higher costs compared with laboratory or clinical parameters [2].

Positron emission tomography (PET), either coupled or not with CT, is an imaging modality that is able to define specific metabolic patterns of cells and tissues by using different radiopharmaceuticals. Its role in oncology has been widely evaluated and its now considered mandatory for the evaluation of a high number of neoplasms [16,17,18,19]. Incidental findings of PET imaging are frequent and therefore need to be clearly evaluated and classified, given the high risk of having false-positive interpretation or masking coexisting pathologies [20,21]. In this context, the bone does not present any exception, and the presence of incidental uptakes related to PD can be frequent.

^18^F-fluorodeoxyglucose ([^18^F]FDG) is the most used radiopharmaceutical for PET imaging, and it has the ability to detect hypermetabolic lesions based on their glycolytic activity. The role of this radiopharmaceutical for the assessment of PD has been widely reported in the literature as bones affected by the disease can express variable degrees of [^18^F]FDG uptake. In this setting, standardized uptake values (SUVmax) ranging from 2.4 to 5.6 were reported, and it is thought that this variability may depend on the presence of lytic or blastic phases of the disease [1,3,6,7,9,14,22,23,24,25,26,27,28,29,30]. However, some articles did not report [^18^F]FDG uptake in PD localization or underline any correlation between osteoblastic and osteoclastic activity with ^18^F-FDG uptake [5,6,8]. Furthermore, it has also been reported that 13% of PD lesions express high [^18^F]FDG uptake, which can mimic the presence of metastatic disease [6]. An example of PD with increased ^18^F-FDG uptake mimicking metastatic disease is shown in Figure 1.

Interestingly, [^18^F]FDG PET can also be useful for the assessment of sarcomatous malignant transformation of PD, and in this setting, PET/CT demonstrated the ability to underline differential intense [^18^F]FDG uptake in transformed areas [31]. However, in contrast with this evidence, the absence of [^18^F]FDG uptake in osteosarcomas arising from PD has also been reported [1]. Malignant transformation to sarcoma has been approximately estimated in 1% of patients with longstanding PD, although this phenomenon increases the risk of developing osteosarcoma in pagetic patients 30 times greater than that of the general population of patients over the age of 40. Usually, a single focus of the neoplasm is seen; however, in some cases, multiple foci are observed, which may reflect the independent multicentric origin of the tumor or metastasis from a single lesion [2].

Prostate-specific membrane antigen (PSMA) is a molecule expressed in a high variety of human tissues, and PET/CT with the PSMA ligand, labelled with both ^18^F and ^68^Ga, is usually used for the assessment of patients with prostate cancer. PSMA expression in non-prostatic tumor lesions has been described and is thought to be related to its overexpression in the endothelial cells of neovasculature of neoplastic and non-neoplastic conditions, stimulated by angiogenetic factors [10,13,32,33,34,35]. However, another alternative hypothesis to explain such uptake is that PSMA accumulation may be driven by hyperemia and the subsequent increased delivery of the radiopharmaceutical [33,35]. Similar to what was previously underlined for [^18^F]FDG, incidental findings related to the presence of PD have been reported by radiolabeled-PSMA PET, with variable degrees of radiopharmaceutical uptake [10,11,12,13,27,33,34,35,36,37]. Interestingly, it has been suggested that PSMA expression is related to bone formation and repair and that uptake of PD at PET imaging is associated with increased bone remodeling and vascularization [11,12]. Conventional radiological imaging (such as CT) remains important to distinguish between sclerotic metastatic foci from PD localization, given its ability to evaluate bone expansion and thickening of trabeculae [12,33].

^18^F-sodium fluoride ([^18^F]NaF) is a PET radiopharmaceutical that specifically reflects the blood flow and the osteoblastic activity of the bone, and it is therefore used for the assessment of skeletal metabolism [38]. [^18^F]NaF PET/CT is able to assess both bone metastases and benign alteration, and the increased osteoblastic activity typical of PD can be clearly evaluated by this imaging modality [4,6,8,39,40,41]. Lastly, other positron-emitter radiopharmaceuticals have demonstrated the ability to visualize PD, such as [^11^C]C-choline, [^68^Ga]Ga-DOTANOC, [^18^F]F-fluciclovine and [^11^C]C-acetate [15,36,42,43].

In conclusion, PD is a frequent incidental finding in PET imaging performed with various radiopharmaceuticals. As the degree of uptake of PD lesions can be very heterogeneous, also mimicking the presence of metastases, this evidence has to be taken into account for the interpretation of PET scans in particular when evaluating oncological patients in order to avoid false-positive interpretation (“not all that glitters is gold”) or the masking of metastatic lesions.

## Figures and Tables

**Figure 1:**
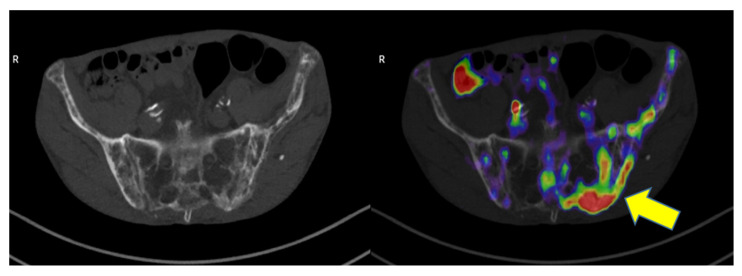
An example of Paget disease in CT (on the **left**) with increased [^18^F]FDG uptake (yellow arrow) at PET/CT (on the **right**).
